# Resting heart rate as an independent predictor of major adverse cardiovascular events in patients receiving maintenance hemodialysis: a prospective cohort study

**DOI:** 10.3389/fmed.2026.1850489

**Published:** 2026-07-30

**Authors:** Ruoyu Tong, Liming Fan, Zhengmao Luo, Zijie Huang, Weidong Zhang, Meng Shen, Qiong Wu, Kairong Zheng, Xinwei Wang, Jin Li, Shuzhen Zhou, Yuanhang Huang, Xianyang Zhong

**Affiliations:** Department of Nephrology, General Hospital of Southern Theater Command, People's Liberation Army, Guangzhou, China

**Keywords:** cardiovascular risk prediction, maintenance hemodialysis, major adverse cardiovascular events (MACE), nomogram, resting heart rate

## Abstract

**Background:**

Existing clinical evidence in the literature regarding the association between resting heart rate (RHR) and major adverse cardiovascular events (MACE) in patients receiving maintenance hemodialysis (MHD) remains unclear and inconsistent.

**Objective:**

To determine whether RHR independently predicts major adverse cardiovascular events (MACE) in patients receiving maintenance hemodialysis (MHD).

**Methods:**

We conducted a prospective cohort study in which 309 adult MHD patients receiving maintenance hemodialysis were stratified into three groups according to the 33rd and 66th percentiles of baseline RHR. The primary outcome was incidence of MACE. Univariaable and multivariaable logistic regression analyses were performed, followed by internal validation of the final multivariable model using bootstrap resampling. A nomogram, calibration plots, and decision curve analysis (DCA) were used to evaluate model performance and clinical utility. It is noteworthy that heart failure events were identified based on the documented clinical diagnosis made by the treating physician after comprehensive assessment of symptoms, signs, medical history, and available investigations. Although BNP measurements and echocardiographic assessments were not available for all patients, their absence did not preclude a clinical diagnosis of heart failure in routine practice.

**Results:**

The incidence of total MACE increased progressively across RHR tertiles (T1: 20.5%, T2: 38.0%, T3: 52.6%; *p* < 0.001). In the multivariable model, higher RHR (T2: odds ratio [OR] = 2.09; T3: OR = 4.70), higher Hospital Anxiety and Depression Scale (HADS) score (8–10: OR = 6.40; >10: OR = 2.04), diabetes (OR = 6.52), elevated intact parathyroid hormone (iPTH) (OR = 1.001), and lower uric acid (OR = 0.74) were identified as independent predictors of total MACE. The nomogram demonstrated good discrimination (area under the curve [AUC] = 0.820, specificity = 0.843, and sensitivity = 0.652), with good calibration and potential clinical utility demonstrated by calibration plots and DCA.

**Conclusion:**

In this single-center cohort of patients receiving maintenance hemodialysis, elevated RHR was independently associated with an increased risk of MACE after adjustment for psychosocial and metabolic factors, including HADS score, diabetes, uric acid, and iPTH. Incorporating RHR and these clinical variables into a risk model may facilitate early identification of high-risk patients for targeted intervention, although external validation is required before broader clinical application.

## 1 Introduction

Cardiovascular disease (CVD) remains the leading cause of mortality in patients with end-stage kidney disease (ESKD) **receiving maintenance** hemodialysis (MHD), accounting for nearly 40% of all deaths in this population. Unlike in the general population, cardiovascular complications in these patients are driven by a complex interplay of uremic toxins, chronic inflammation, volume overload, autonomic dysfunction, and disturbance in mineral metabolism (1, 2). These mechanisms accelerate atherosclerosis, promote left ventricular hypertrophy, and contribute to myocardial fibrosis, thereby increasing the risk of major adverse cardiovascular events (MACE), including myocardial infarction (MI), stroke, and heart failure (HF) (1–3). Traditional cardiovascular risk factors, including dyslipidemia, diabetes, hypertension, together with vascular complications including atherosclerosis, and coronary artery calcification, also contribute substantially to cardiovascular morbidity among patients with CKD and those receiving MHD (4).

To enhance cardiovascular risk stratification in patients with chronic kidney disease (CKD), several studies have investigated advanced diagnostic modalities and novel prognostic markers. For instance, the Gunma-CKD SPECT studies demonstrated that myocardial perfusion imaging parameters, such as the summed difference score (SDS), together with cardiac functional and clinical parameters, including left ventricular ejection fraction (LVEF), and estimated glomerular filtration rate (eGFR), can predict cardio-cerebrovascular and renal outcomes, highlighting the potential value of advanced imaging-based assessments for cardiovascular risk stratification (5, 6). However, despite their potential value, such approaches are not routinely available in many dialysis cohorts due to resource requirements and limited feasibility in routine clinical practice. Moreover, these studies primarily involved non-dialysis CKD populations and did not account for risk factors specific to MHD patients, such as autonomic imbalance and psychosocial burden (7). Therefore, identifying simpler, readily accessible markers that can be easily integrated into routine clinical practice remains clinically important.

Resting heart rate (RHR), by contrast, represents is a simple, non-invasive and routinely available clinical parameter that reflects autonomic regulation and has been increasingly recognized as a prognostic marker in the general population and in heart failure (HF) cohorts. Given the prominent role of autonomic dysfunction in MHD patients, RHR may provide a practical approach for cardiovascular risk assessment in this high-risk population (8–10). In MHD patients, elevated RHR may reflect heightened sympathetic activity and impaired autonomic balance, which can result from common dialysis-related conditions, including anemia, intermittent volume shifts, and metabolic disturbances (11–13). Despite its clinical simplicity and established prognostic relevance in other populations, the prognostic value of RHR in predicting MACE in MHD patients remains unclear. Although elevated RHR has been associated with mortality in dialysis populations (14, 15), evidence directly linking it to major adverse cardiovascular events (MACE) is limited and inconsistent.

To address these gaps, we conducted a prospective cohort study of 309 patients receiving MHD to determine whether baseline RHR independently predicts MACE after adjustment for relevant psychosocial, clinical, and biochemical factors. We also developed and internally validated a nomogram incorporating RHR and other independent predictors to facilitate individualized cardiovascular risk prediction in this population.

## 2 Materials and methods

### 2.1 Study design and participants

This prospective cohort study enrolled 309 patients receiving MHD at the Hemodialysis Center, General Hospital, the Southern Theater Command, Chinese People’s Liberation Army, between January 2018 and January 2024.

The inclusion criteria were: (1) age ≥18 years; and (2) end-stage kidney disease (ESKD) treated with maintenance hemodialysis (2–3 sessions per week, 4 h per session) for at least 3 months. The exclusion criteria were: (1) connective tissue disease or treatment with immunosuppressive agents; (2) malignancy; (3) active systemic infection; (4) prior peritoneal dialysis or kidney transplantation; and (5) incomplete clinical data.

All patients underwent bicarbonate-based hemodialysis with blood flow rates of 200–350 mL/min and a dialysate flow rate of 500 mL/min. Anticoagulation was achieved using either unfractionated heparin or low-molecular-weight heparin according to each patients’ coagulation status. Vascular access was provided via an arteriovenous fistula or a central venous catheter.

Participants were followed prospectively from study enrollment until the occurrence of the first MACE event or January 2024, whichever came first. The median follow-up duration was 22.1 months, and no patients were lost to follow-up.

### 2.2 Data collection and variables

Baseline data were collected 3 months after initiation of hemodialysis to minimize the influence of early dialysis-related instability. Data collected included demographic characteristics (age, sex, dialysis duration), comorbidities (diabetes, hypertension, prior stroke), medication use (including *β*-blocker therapy), and laboratory parameters. Laboratory measurements included hemoglobin, hematocrit, serum albumin, glycated hemoglobin (HbA1c), high-sensitivity C-reactive protein (hs-CRP), calcium, corrected calcium, phosphate, calcium–phosphate product, Kt/V, uric acid (UA), lipid profile (triglycerides, total cholesterol, high-density lipoprotein cholesterol [HDL-C], low-density lipoprotein cholesterol [LDL-C]), and intact parathyroid hormone (iPTH). Resting heart rate (RHR) was measured using a standard 12-lead electrocardiogram before dialysis session after at least 20 min of supine rest. Psychological status was assessed using the Hospital Anxiety and Depression Scale (HADS), a validated 14-item questionnaire consisting of anxiety and depression subscales, each scored 0–21. All clinical data were independently reviewed by two investigators for accuracy.

Smoking status, atrial fibrillation history, residual renal function, and detailed echocardiographic parameters (including LVH and LVEF) were not systematically available and were therefore not included in the analyses. Because *β*-blockers may influence resting heart rate, β-blocker use was included as a covariate in the analyses. Other medications were not included in multivariable analyses because their use may have reflected underlying disease severity and introduced potential confounding by indication.

### 2.3 RHR group stratification and outcomes

Patients were stratified into tertiles according to baseline RHR to evaluate the relationship between different RHR levels and the risk of MACE. The groups were defined as: T1 (≤71 bpm), T2 (72–83 bpm), and T3 (≥84 bpm).

The primary outcome was major adverse cardiovascular events (MACE), defined as a composite of fatal or non-fatal myocardial infarction, fatal or non-fatal stroke, and hospitalization for heart failure. Myocardial infarction was defined according to the Fourth Universal Definition (2018). Stroke events were confirmed by computed tomography or magnetic resonance imaging.

Heart failure (HF) events were identified based on clinical diagnoses documented by treating physicians, supported by relevant symptoms, physical findings, imaging findings when available, and laboratory evidence (27).

### 2.4 Statistical analysis

Missing data were observed only for corrected calcium (7.8%), hs-CRP (13.9%), and hematocrit (43.7%). Because of incomplete data availability, these variables were included only in univariable analyses, and no imputation was performed.

All analyses were performed using R software (version 4.4.2). Continuous variables were assessed for normality using the Kolmogorov–Smirnov test and presented as median (IQR) because of non-normal distributions. Baseline characteristics across RHR tertiles were compared using the Kruskal–Wallis test with post-hoc Dunn tests for continuous variables and chi-square or Fisher’s exact test for categorical variables, as appropriate.

Univariable logistic regression analyses were performed to evaluate candidate predictors of MACE. Variables considered clinically relevant or statistically significant in univariable analyses were entered into the multivariable logistic regression model. Logistic regression was selected because the primary objective was to develop a baseline risk prediction model rather than a time-to-event model. Multicollinearity was assessed using variance inflation factor (VIF).

A nomogram was constructed based on the final multivariable logistic regression model. Model discrimination was assessed using receiver operating characteristic (ROC) curve analysis, including the area under the curve (AUC), sensitivity, and specificity. Calibration was evaluated using calibration plots to assess agreement between predicted and observed probabilities. Internal validation was performed using bootstrap resampling with 1,000 iterations. Decision curve analysis (DCA) was conducted to evaluate the clinical utility and net benefit of the model. A two-sided *p* value <0.05 was considered statistically significant.

## 3 Results

### 3.1 Baseline clinical characteristics according to resting heart rate groups

A total of 309 patients were included in this study. Based on baseline resting heart rate (RHR), patients were categorized into three groups: T1 (n = 112), T2 (n = 100), and T3 (n = 97). The cohort comprised 185 (59.9%) men and 124 (40.1%) women, with a median age of 56 years (IQR: 40–67). Baseline characteristics according to RHR group are summarized in Table 1.

**Table 1:** Baseline clinical characteristics according to resting heart rate (RHR) group.

Parameters	T1 (*n* = 112)	T2 (*n* = 100)	T3 (*n* = 97)	*P*
Sex				0.686
Male	64 (57.14%)	63 (63.00%)	58 (59.79%)	
Female	48 (42.86%)	37 (37.00%)	39 (40.21%)	
Age, year	59 (41.25, 67)	55 (43, 64.50)	56 (38, 69.10)	0.630
Age group				0.269
<65	73 (65.18%)	75 (75.00%)	65 (67.01%)	
≥65	39 (34.82%)	25 (25.00%)	32 (32.99%)	
BMI, kg/m^2^	21.56 (20.08, 24.03)	21.62 (19.78, 23.05)	20.34 (17.78, 22.63) ^ab^	0.001
BMI group				<0.001
<18.5	7 (6.25%)	9 (9.00%)	29 (29.90%)	
18.5 to 24.0	89 (79.46%)	79 (79.00%)	58 (59.79%)	
>24.0	16 (14.29%)	12 (12.00%)	10 (10.31%)	
Residence				0.335
Rural	35 (31.25%)	24 (24.00%)	32 (32.99%)	
City	77 (68.75%)	76 (76.00%)	65 (67.01%)	
Disease cause				0.158
Nephritis	43 (38.39%)	36 (36.00%)	23 (23.71%)	
DKD	29 (25.89%)	36 (36.00%)	43 (44.33%)	
HKD	12 (10.71%)	7 (7.00%)	6 (6.19%)	
Obstructive nephropathy	6 (5.36%)	5 (5.00%)	8 (8.25%)	
Others	22 (19.64%)	16 (16.00%)	17 (17.53%)	
HADS score	10.50 (6, 13.75)	9 (6, 13)	7 (5, 12) ^ab^	0.012
HADS group				0.035
≤7	48 (42.86%)	45 (45.00%)	53 (54.64%)	
8 to 10	8 (7.14%)	16 (16.00%)	14 (14.43%)	
10 < to ≤21	56 (50.00%)	39 (39.00%)	30 (30.93%)	
Disease history				
Diabetes	22 (19.64%)	26 (26.00%)	29 (29.90%)	0.222
Hypertension	59 (52.68%)	53 (53.00%)	48 (49.48%)	0.860
CVD	35 (31.25%)	30 (30.00%)	27 (27.84%)	0.863
CBD	8 (7.14%)	10 (10.00%)	12 (12.37%)	0.441
Using β blocker	30 (26.79%)	23 (23.00%)	39 (40.21%)	0.021
RHR	69 (67, 71)	78 (76, 78) ^a^	88 (83.50, 95) ^ab^	<0.001
eGFR	7.78 (6.35, 9.65)	7.78 (6.78, 9.29)	7.78 (5.68, 8.71)	0.219
SBP	139.50 (130, 150)	145 (130, 152.75)	140 (126, 150)	0.161
DBP	80 (75.25, 90)	80 (75.25, 90)	80 (75, 88)	0.800
MAP (before dialysis)	103 (95.08, 108.33)	103.33 (95.67, 109.25)	103 (93.33, 107)	0.512
Laboratory results				
Serum Ca, mg/dL	8.95 (8.34, 9.49)	8.97 (8.47, 9.33)	9.04 (8.51, 9.36)	0.842
Corrected Ca	9.23 (8.70, 9.68)	9.26 (8.73, 9.60)	9.26 (8.94, 9.63)	0.525
Serum P, mg/dL	5.13 (4.34, 5.73)	5.16 (4.54, 6.11)	4.87 (4.08, 5.92)	0.181
Ca × P	44.54 (38.05, 52.04)	45.55 (39.16, 54.43)	45.50 (36.74, 54.55)	0.548
Kt/V	2.02 (1.38, 2.17)	1.98 (1.41, 2.17)	1.75 (1.43, 2.17)	0.777
HB	99.50 (87.18, 113.14)	100 (85.55, 110.70)	95 (85, 109.59)	0.413
ALB	37 (34.48, 39.55)	36.49 (33.63, 39.44)	36.44 (33.58, 39.58)	0.534
hs-CRP	4.96 (1.47, 6.26)	5.55 (2.97, 6.26)	4.32 (1.29, 6.26)	0.150
UA, mg/dL	7.01 (6.01, 8.11)	6.91 (5.91, 7.58)	6.95 (6.14, 7.63)	0.314
TC	4.73 (3.98, 5.32)	4.89 (3.94, 5.67)	4.89 (3.93, 5.46)	0.821
TG	1.45 (1.18, 2.15)	1.56 (1.10, 2.21)	1.46 (1.05, 1.90)	0.733
LDL-C	1.29 (1.02, 1.60)	1.23 (1.06, 1.74)	1.37 (1.03, 1.70)	0.863
HDL-C	2.53 (2.09, 3.22)	2.53 (2.00, 3.11)	2.56 (1.97, 3.18)	0.823
HCT	28 (24.87, 33.26)	26.43 (22.48, 32.13)	25.73 (22.94, 30.14)	0.131
Ferritin	345 (223.29, 543)	321 (204.89, 561)	311.70 (173.18, 454.50)	0.132
iPTH, pg./mL	158.25 (98.51, 410.44)	220.65 (124.84, 455.75)	234 (125.08, 455)	0.075

^a^*P* < 0.05 compared to T1 group; ^b^*P* < 0.05 compared to T2 group. Patients were categorized into three groups based on RHR tertiles at baseline: T1 (≤33rd percentile, ≤71 bpm), T2 (33rd–66th percentile, 72–83 bpm), T3 (>66th percentile, ≥84 bpm). ALB, albumin; BMI, body mass index; Ca, calcium; Ca × P, calcium-phosphorus product; CBD, cerebrovascular disease; CVD, cardiovascular disease; DBP, diastolic blood pressure; DKD, diabetic kidney disease; eGFR, estimated glomerular filtration rate; HADS, Hospital Anxiety and Depression Scale; HB, hemoglobin; HCT, hematocrit; HDL-C, high-density lipoprotein cholesterol; HKD, hypertensive kidney disease; hs-CRP, high-sensitivity C-reactive protein; iPTH, intact parathyroid hormone; Kt/V, dialysis adequacy index (dialyzer clearance of urea × dialysis time / volume of distribution of urea); LDL-C, low-density lipoprotein cholesterol; MAP, mean arterial pressure; P, phosphorus; RHR, resting heart rate; SBP, systolic blood pressure; TC, total cholesterol; TG, triglyceride; UA, uric acid.

Compared with patients in the T1 and T2 groups, Patients in the T3 group had significantly lower body mass index (BMI) and Hospital Anxiety and Depression Scale (HADS) scores and a higher rate of *β*-blocker use (all *p* < 0.05). No significant between-group differences were observed in age, sex, comorbidities, blood pressure, or most laboratory parameters (Table 1).

### 3.2 Mortality and MACE outcomes

All enrolled patients completed follow-up. The median follow-up duration was 22.1 months (IQR: 10.5–39.4 months), corresponding to 584.13 person-years of follow-up. No patients were lost to follow-up. The incidence of MACE increased progressively across the three RHR groups (T1: 20.5%, T2: 38.0%, T3: 52.6%; *p* < 0.001) (Table 2).

**Table 2:** Incidence of major adverse cardiovascular events (MACE) and other clinical outcomes according to resting heart rate (RHR) group.

Parameters	T1 (*n* = 112)	T2 (*n* = 100)	T3 (*n* = 97)	*P*
MI	6 (5.36%)	11 (11.00%)	13 (13.40%)	0.128
Stroke	7 (6.25%)	7 (7.00%)	17 (17.53%)	0.012
HF hospitalization	10 (8.93%)	20 (20.00%)	21 (21.65%)	0.025
Total MACE	23 (20.54%)	38 (38.00%)	51 (52.58%)	<0.001
Other clinical outcomes				
CVD event	16 (14.29%)	31 (31.00%)	33 (34.02%)	0.002
PAD	12 (10.71%)	12 (12.00%)	29 (29.90%)	<0.001
Fracture	10 (8.93%)	10 (10.00%)	18 (18.56%)	0.075
IDH	20 (17.86%)	21 (21.00%)	31 (31.96%)	0.045

Patients were categorized into three groups based on RHR tertiles at baseline: T1 (≤33rd percentile, ≤71 bpm), T2 (33rd–66th percentile, 72–83 bpm), T3 (>66th percentile, ≥84 bpm). CVD, cardiovascular disease; HF, heart failure; IDH, intradialytic hypotension; MACE, major adverse cardiovascular events; MI, myocardial infarction; PAD, peripheral artery disease; RHR, resting heart rate.

All pre-specified outcome variables (MACE components: myocardial infarction, stroke, and heart failure hospitalization) were completely ascertained for all participants. No outcome data were missing.

### 3.3 Comparison between patients with or without MACE

Table 3 summarizes baseline characteristics according to MACE status.

**Table 3:** Comparison of clinical characteristics between patients with or without major adverse cardiovascular events (MACE).

Parameters	Non-MACE (*n* = 197)	Total MACE (*n* = 112)	All (*n* = 309)	*P*
RHR				<0.001
T1 (≤33rd percentile)	89 (45.18%)	23 (20.54%)	112 (36.25%)	
T2 (33rd–66th percentile)	62 (31.47%)	38 (33.93%)	100 (32.36%)	
T3 (>66th percentile)	46 (23.35%)	51 (45.54%)	97 (31.39%)	
Sex				0.820
Male	117 (59.39%)	68 (60.71%)	185 (59.87%)	
Female	80 (40.61%)	44 (39.29%)	124 (40.13%)	
Age, year	55 (38.50, 67)	57 (45.50, 68)	56 (40, 67.11)	0.185
Age group				0.758
<65	137 (69.54%)	76 (67.86%)	213 (68.93%)	
≥65	60 (30.46%)	36 (32.14%)	96 (31.07%)	
BMI, kg/m^2^	21.11 (19.34, 23.05)	21.36 (19.60, 23.47)	21.28 (19.38, 23.18)	0.231
BMI group				0.296
<18.5	33 (16.75%)	12 (10.71%)	45 (14.56%)	
18.5 to 24.0	142 (72.08%)	84 (75.00%)	226 (73.14%)	
>24.0	22 (11.17%)	16 (14.29%)	38 (12.30%)	
Residence				0.997
Rural	58 (29.44%)	33 (29.46%)	91 (29.45%)	
City	139 (70.56%)	79 (70.54%)	218 (70.55%)	
Disease cause				<0.001
Nephritis	82 (41.62%)	20 (17.86%)	102 (33.01%)	
DKD	36 (18.27%)	72 (64.29%)	108 (34.95%)	
HKD	25 (12.69%)	0 (0.00%)	25 (8.09%)	
Obstructive nephropathy	9 (4.57%)	10 (8.93%)	19 (6.15%)	
Others	45 (22.84%)	10 (8.93%)	55 (17.80%)	
HADS score	7 (6, 13)	9 (6, 13)	8 (6, 13)	0.546
HADS group				0.002
≤7	102 (51.78%)	44 (39.29%)	146 (47.25%)	
8 to 10	15 (7.61%)	23 (20.54%)	38 (12.30%)	
10 < to ≤21	80 (40.61%)	45 (40.18%)	125 (40.45%)	
Disease history				
Diabetes	25 (12.69%)	52 (46.43%)	77 (24.92%)	<0.001
Hypertension	89 (45.18%)	71 (63.39%)	160 (51.78%)	0.002
CVD	56 (28.43%)	36 (32.14%)	92 (29.77%)	0.492
CBD	18 (9.14%)	12 (10.71%)	30 (9.71%)	0.653
Using β blocker	52 (26.40%)	40 (35.71%)	92 (29.77%)	0.085
RHR	76 (69, 80)	78.50 (74, 88)	77 (69, 83)	<0.001
SBP	140 (130, 150)	141 (130, 150)	140 (130, 150)	0.664
DBP	82 (76, 90)	80 (75, 87)	80 (75, 90)	0.025
MAP (before dialysis)	103.33 (95.50, 109.50)	103 (93.33, 106.25)	103.33 (95, 108.33)	0.158
Laboratory results				
Serum Ca, mg/dL	8.93 (8.38, 9.35)	9.07 (8.50, 9.43)	8.97 (8.40, 9.40)	0.146
Corrected Ca	9.20 (8.75, 9.58)	9.38 (8.88, 9.72)	9.25 (8.78, 9.64)	0.028
Serum P, mg/dL	5.02 (4.35, 5.87)	5.22 (4.16, 5.92)	5.08 (4.34, 5.91)	0.907
Ca × P	44.53 (38.29, 53.71)	45.49 (37.89, 55.09)	44.89 (38.29, 54.08)	0.462
Kt/V	1.89 (1.35, 2.17)	1.89 (1.45, 2.17)	1.89 (1.42, 2.17)	0.949
HB	99.47 (85.75, 111.83)	96.92 (86.38, 109.44)	99.25 (86, 110.63)	0.586
ALB	36.60 (34, 39.68)	36.76 (33.83, 39.33)	36.70 (33.98, 39.50)	0.674
hs-CRP	4.29 (1.66, 6.26)	6.26 (2.54, 6.26)	4.85 (1.97, 6.26)	0.045
UA, mg/dL	7.01 (6.29, 7.87)	6.74 (5.58, 7.40)	6.96 (6.04, 7.73)	0.002
TC	4.67 (3.93, 5.37)	4.89 (3.97, 5.64)	4.87 (3.94, 5.42)	0.140
TG	1.45 (1.12, 1.99)	1.67 (1.15, 2.24)	1.48 (1.12, 2.13)	0.044
LDL-C	1.29 (1.00, 1.62)	1.31 (1.05, 1.71)	1.31 (1.03, 1.67)	0.517
HDL-C	2.53 (1.95, 3.11)	2.55 (2.03, 3.21)	2.53 (2.01, 3.18)	0.414
HCT	26.45 (23.72, 32.61)	26.72 (23.32, 31.16)	26.53 (23.44, 31.58)	0.686
Ferritin	344 (228.30, 543)	265.75 (173.47, 455.25)	335.90 (212.37, 470.85)	0.009
iPTH, pg./mL	167.40 (120, 345)	266.28 (128.99, 513.30)	222 (122.68, 432)	0.002

ALB, albumin; BMI, body mass index; Ca, calcium; Ca × P, calcium–phosphorus product; CBD, cerebrovascular disease; CVD, cardiovascular disease; DBP, diastolic blood pressure; DKD, diabetic kidney disease; HADS, Hospital Anxiety and Depression Scale; HB, hemoglobin; HCT, hematocrit; HDL-c, high-density lipoprotein cholesterol; HKD, hypertensive kidney disease; Hs-CRP, high-sensitivity C-reactive protein; iPTH, intact parathyroid hormone; Kt/V, dialysis adequacy index (dialyzer clearance of urea × dialysis time / volume of distribution of urea); LDL-C, low-density lipoprotein cholesterol; MACE, major adverse cardiovascular events; MAP, mean arterial pressure; P, phosphorus; RHR, resting heart rate; SBP, systolic blood pressure; TC, total cholesterol; TG, triglycerides; UA, uric acid.

Patients with MACE had significantly higher RHR, a greater prevalence of diabetic kidney disease, and higher HADS scores, with a higher proportion of moderate psychological symptoms. Diabetes and hypertension were also more prevalent among patients who experienced MACE.

Regarding clinical and laboratory parameters, the MACE group had lower DBP, UA, and ferritin levels but higher corrected calcium, triglycerides, iPTH, and hs-CRP (all *p* < 0.05).

### 3.4 Independent predictors of total MACE

Univariable logistic regression analysis identified variables associated with total MACE (Table 4). In the multivariable analysis, higher RHR (T2: OR = 2.09, *p* = 0.041; T3: OR = 4.70, *p* < 0.001), higher HADS scores (8–10: OR = 6.40, *p* < 0.001; >10: OR = 2.04, *p* = 0.028), diabetes (OR = 6.52, *p* < 0.001), elevated iPTH (OR = 1.15, *p* = 0.002), and lower UA levels (OR = 0.74, *p* = 0.002) remained independently associated with MACE (Table 4). Hypertension and DBP were not significant after multivariable adjustment. No significant multicollinearity was observed among the included predictors (all VIF 1.02–1.09).

**Table 4:** Logistic regression analysis of independent predictors of total major adverse cardiovascular events (MACE).

Parameters	Univariable		Multivariable		
	OR (95% CI)	*P*	OR (95% CI)	*P*	VIF
RHR		<0.001		<0.001	1.02
T1 (≤33rd percentile)	1	–	1	–	
T2 (33rd–66th percentile)	2.37 (1.29 to 4.37)	0.006	2.09 (1.03 to 4.25)	0.041	
T3 (>66th percentile)	4.29 (2.34 to 7.88)	<0.001	4.70 (2.27 to 9.73)	<0.001	
HADS		0.004		<0.001	1.04
≤7	1	–	1	–	
8 to 10	3.55 (1.70 to 7.45)	<0.001	6.40 (2.65 to 15.48)	<0.001	
10< to ≤21	1.30 (0.78 to 2.17)	0.306	2.04 (1.08 to 3.84)	0.028	
Diabetes					1.09
No	1	–	1	–	
Yes	5.96 (3.41 to 10.44)	<0.001	6.52 (3.30 to 12.86)	<0.001	
Hypertension					1.05
No	1	–	1	–	
Yes	2.10 (1.31 to 3.38)	0.002	1.73 (0.96 to 3.13)	0.070	
DBP	0.98 (0.96 to 1.00)	0.048	0.99 (0.97 to 1.02)	0.682	1.05
UA, mg/dL	0.74 (0.63 to 0.89)	<0.001	0.74 (0.61 to 0.91)	0.004	1.02
TG	1.31 (1.01 to 1.69)	0.040	1.13 (0.82 to 1.57)	0.454	1.03
iPTH, 100 pg./mL	1.12 (1.04 to 1.21)	0.002	1.15 (1.05 to 1.26)	0.002	1.04

DBP, diastolic blood pressure; HADS, Hospital Anxiety and Depression Scale; iPTH, intact parathyroid hormone; MACE, major adverse cardiovascular events; OR, odds ratio; RHR, resting heart rate; TG, triglycerides; UA, uric acid. All baseline variables presented in Table 1 were initially evaluated using univariable logistic regression. Variables that were statistically significant in the univariable analyses were subsequently entered into the multivariable logistic regression model; the results of both analyses are presented in Table 4.

### 3.5 Nomogram development and predictive performance

**Figure 1:**
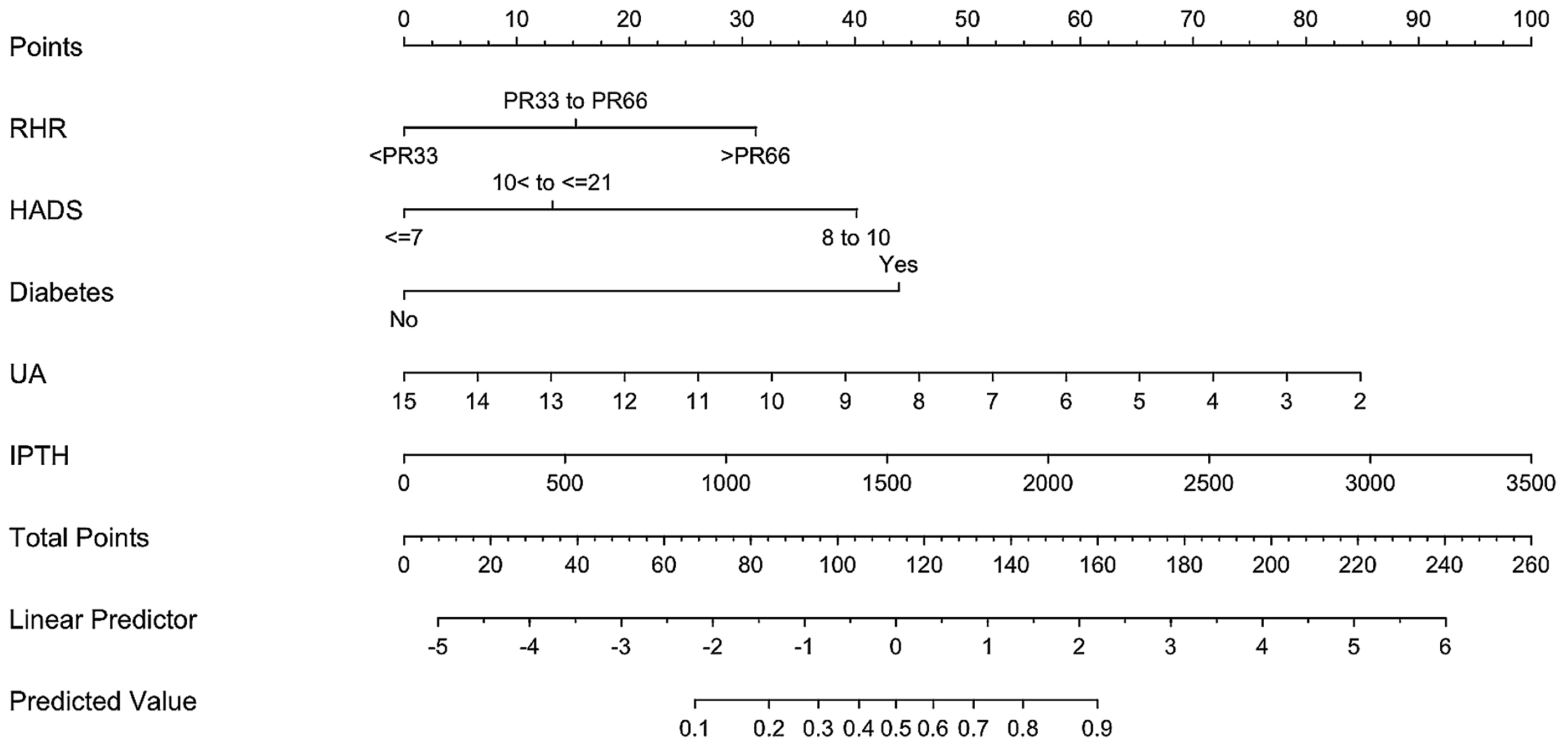
Nomogram based on the multivariable logistic regression model. Each predictor (RHR group, HADS group, diabetes, UA, iPTH) contributes a point score; and total points correspond to the predicted probability of total MACE. RHR: Resting heart rate; HADS: Hospital Anxiety and Depression Scale; UA: Uric acid; iPTH: Intact parathyroid hormone.

Figure 1 illustrates the nomogram based on the final multivariable model.

**Figure 2:**
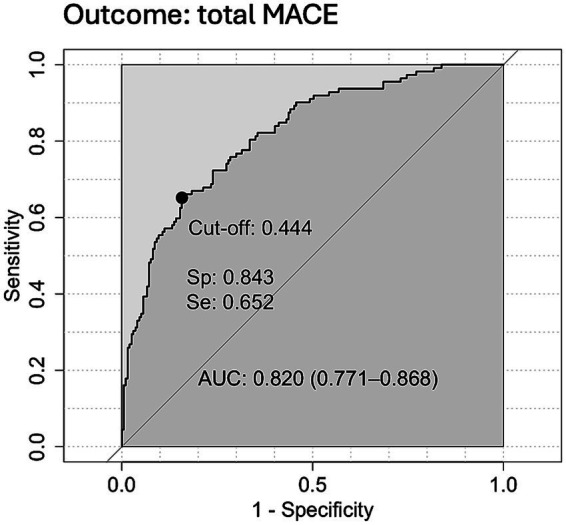
Receiver operating characteristic (ROC) curve of the multivariable logistic regression model for predicting total major adverse cardiovascular events (MACE). The area under the ROC curve (AUC) was 0.820 (95% confidence interval [CI]: 0.771–0.868). The black dot indicates the optimal cut-off value determined by the Youden index (cut-off = 0.444), corresponding to a sensitivity (Se) of 0.652 and specificity (Sp) of 0.843. The diagonal gray line represents the reference line of no discrimination (AUC = 0.5). MACE: Major adverse cardiovascular events; ROC: Receiver operating characteristic; AUC: Area under the curve; CI: Confidence interval; Se: Sensitivity; Sp: Specificity.

The nomogram demonstrated good discrimination with an AUC of 0.820. At the optimal cutoff value, the model achieved a sensitivity of 0.652 and specificity of 0.843 (Figure 2). Calibration analysis showed good agreement between predicted and observed risks, and decision curve analysis (DCA) indicated potential clinical utility.

### 3.6 Internal validation of the multivariable model

**Figure 3:**
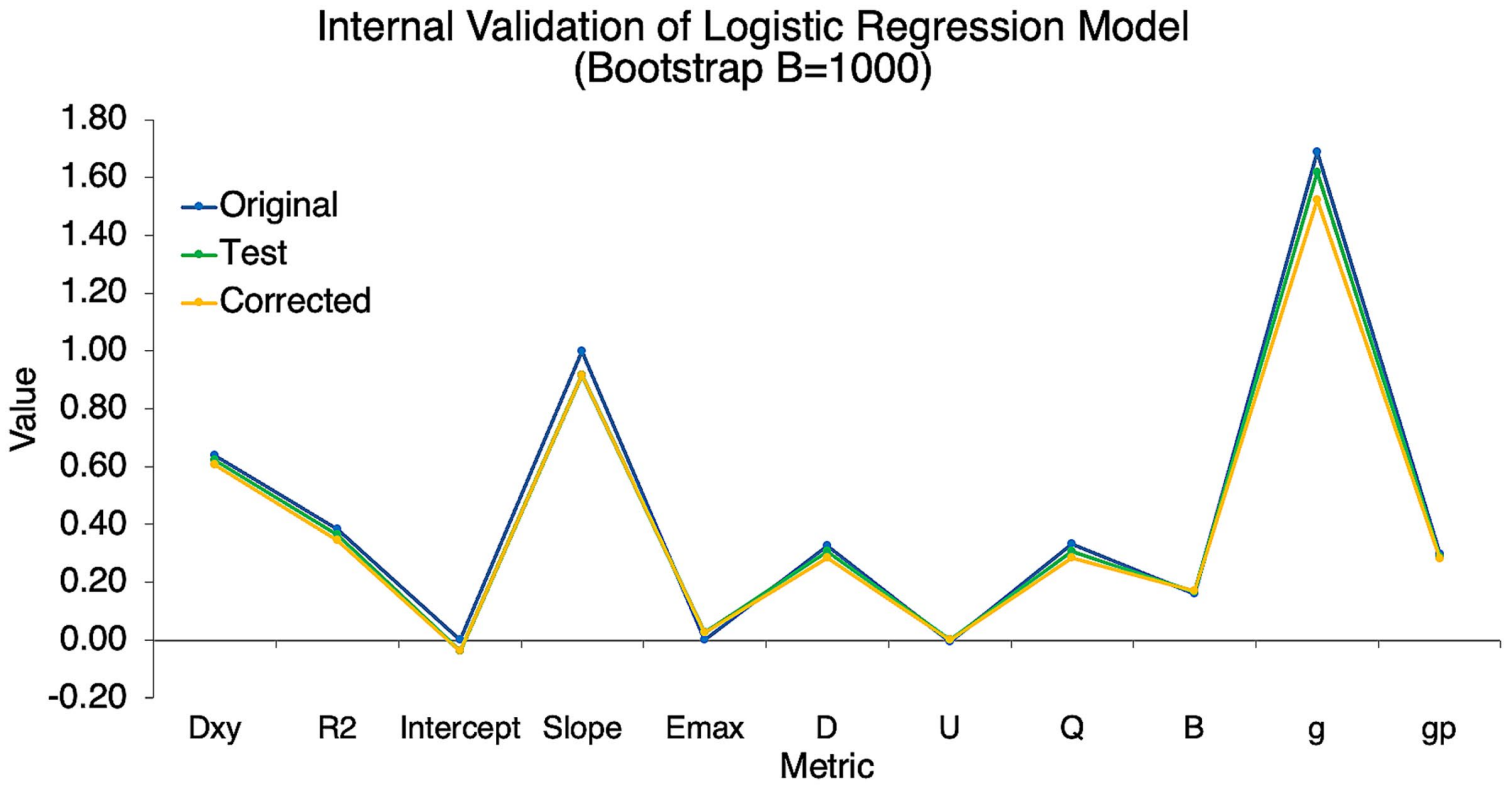
Internal validation of the multivariable logistic regression model using bootstrap resampling (*B* = 1,000). The figure compares the apparent (blue), bootstrap test (green), and optimism-corrected (yellow) performance estimates across multiple validation metrics. *Dxy* represents Somers’ rank correlation; *R*^2^ represents explained variation; intercept and slope reflect calibration performance; *Emax* indicates the maximum calibration error; *D*, *U*, and *Q* are components of the Brier score decomposition; *B* represents the Brier score; *g* and *gp* are discrimination indices related to model prediction performance.

**Figure 4:**
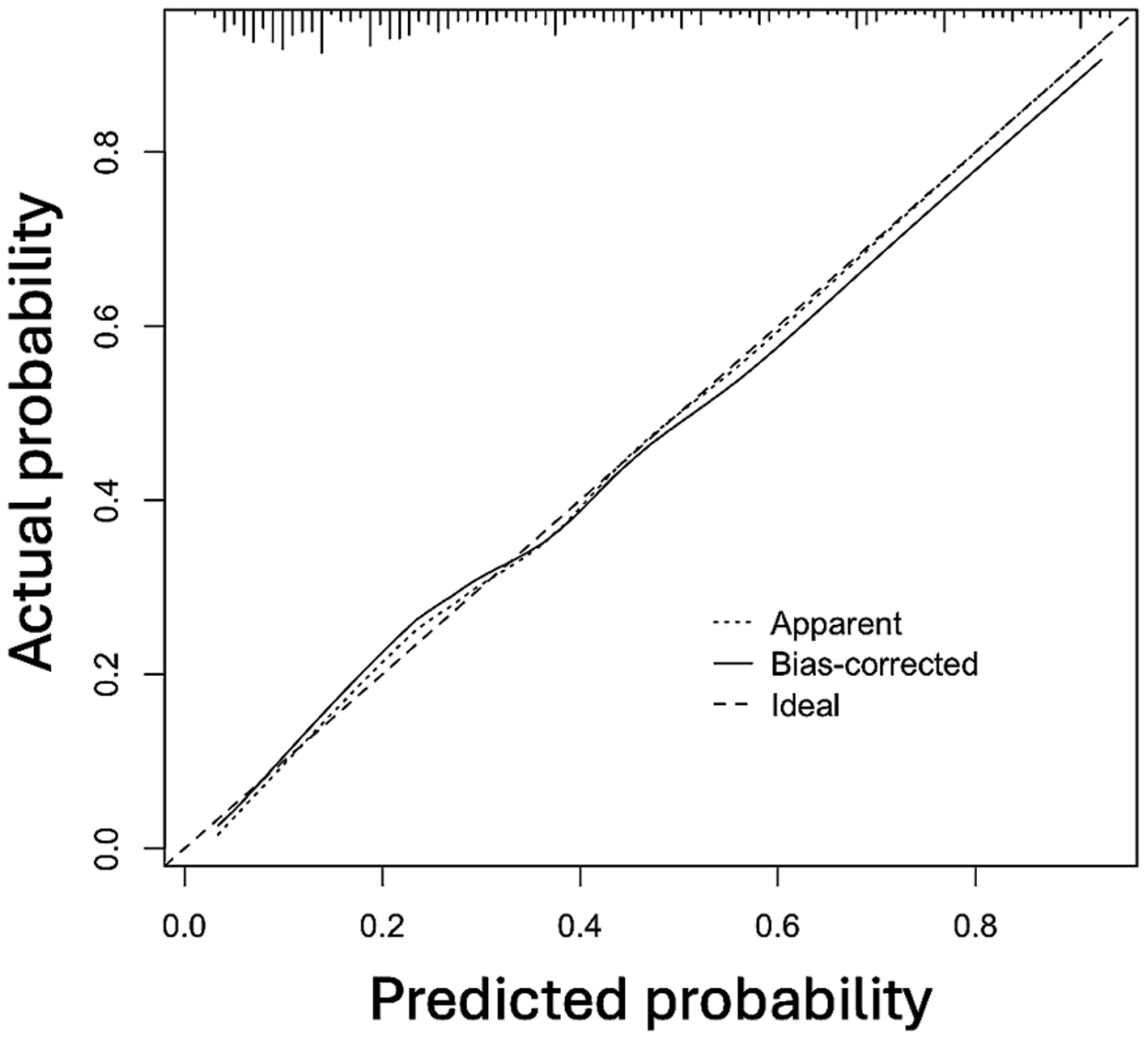
Calibration plot of the multivariable logistic regression model for predicting total major adverse cardiovascular events (MACE). The *x*-axis represents the predicted probability of MACE, and the *y*-axis represents the observed probability. The dashed diagonal line represents the ideal calibration line, indicating perfect agreement between predicted and observed probabilities. The dotted line represents the apparent calibration of the original model, and the solid line represents the bootstrap bias-corrected calibration curve based on 1,000 resamples. MACE: Major adverse cardiovascular events.

Bootstrap resampling (*B* = 1,000) was performed for internal validation of the prediction model. The model showed good stability after bootstrap correction, with only minimal decreases in Somers’ Dxy and *R*^2^. The optimism-corrected AUC was 0.801, with only a small reduction compared with the apparent AUC, suggesting limited overfitting (Table 5 and Figure 3). Calibration plots demonstrated good agreement between predicted and observed probabilities after validation (Figure 4).

**Table 5:** Internal validation of the multivariable logistic regression model using bootstrap resampling.

Metric	Original	Test	Corrected
Dxy	0.64	0.62	0.61
*R* ^2^	0.38	0.37	0.35
Intercept	0.00	−0.04	−0.04
Slope	1.00	0.92	0.92
Emax	0.00	0.03	0.03
D	0.33	0.31	0.29
U	−0.01	0.00	0.00
Q	0.33	0.31	0.28
B	0.16	0.17	0.17
g	1.69	1.62	1.52
gp	0.30	0.29	0.28

B, Brier score; D, discrimination index; Dxy, Somers’ Dxy rank correlation between predicted probabilities and observed binary outcomes; Emax, maximum absolute calibration error; gp, Gini’s mean difference based on predicted probabilities; g, Gini’s mean difference; Intercept, calibration intercept; Q, overall model quality index; R^2^, Nagelkerke’s R-squared; Slope, calibration slope; U, unreliability index.

### 3.7 Decision curve analysis and clinical utility of the predication model

**Figure 5:**
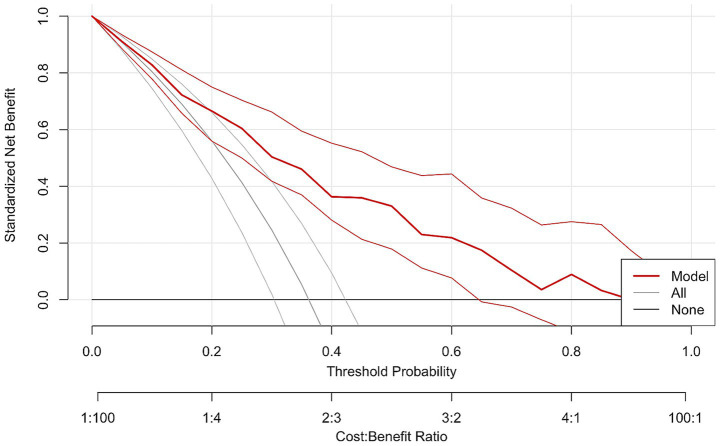
Decision curve analysis (DCA) of the multivariable logistic regression model for predicting total major adverse cardiovascular events (MACE). The *x*-axis represents the threshold probability, and the *y*-axis represents the standardized net benefit. The red line represents the prediction model, the gray line (“All”) represents the strategy of treating all patients as high risk, and the black horizontal line (“None”) represents the strategy of treating no patients as high risk. The lower *x*-axis shows the corresponding cost-to-benefit ratios for different threshold probabilities. DCA: Decision curve analysis; MACE: Major adverse cardiovascular events.

Decision curve analysis demonstrated that the prediction model provided a net clinical benefit over a range of threshold probabilities (approximately 0.32–0.46) compared with treat-all or treat-none strategies (Figure 5).

## 4 Discussion

In this single-center cohort of patients undergoing MHD, a higher RHR was independently associated with an increased risk of MACE after adjustment for traditional cardiovascular risk factors, mineral metabolism markers, and psychological parameters such as depression and anxiety. These findings suggest RHR may serve as a simple, inexpensive, and readily available prognostic marker for cardiovascular risk stratification in patients receiving MHD. Nevertheless, cardiovascular risk in the CKD population is multifactorial, with established determinants such as dyslipidemia, atherosclerotic burden, metabolic syndrome, and coronary artery calcification remaining important contributors to MACE. Thus, RHR should be considered as a complementary marker that provides additional prognostic information beyond conventional risk factors.

Previous studies examining the prognostic significance of RHR in CKD or dialysis populations have reported inconsistent results, partly due to differences in study design, patient characteristics, RHR definitions, and outcome measures. For example, Inaguma et al. reported a RHR ≥ 101 bpm at dialysis initiation was associated with mortality among incident dialysis patients; however, the study relied on a single baseline measurement and did not account for potential psychological or inflammatory contributors (14). Similarly, Cice et al. reported an RHR > 85 bpm was associated with increased cardiac events in MHD patients, although patients with pre-existing cardiovascular disease were excluded and MACE-specific outcomes were not assessed (15). In contrast, our analysis evaluated the association between baseline RHR and MACE using percentile-based RHR categories and multivariable adjustment incorporating clinical, biochemical, and psychological factors, with internal validation of the prediction model. These findings provide additional evidence supporting the prognostic relevance of RHR in patients undergoing MHD.

Importantly, the association between higher RHR and MACE remained significant after adjustment for psychological distress assessed by the HADS. Psychological symptoms are common among patients undergoing MHD, and have been independently associated with cardiovascular risk in CKD (7, 16–18). Our findings indicate that RHR provides prognostic information beyond psychological status alone. Notably, although HADS remained an independent predictor of MACE in the multivariable model, its inclusion did not attenuate the association between RHR and cardiovascular outcomes, suggesting that these factors may contribute to risk through partially independent processes. Future studies incorporating heart rate variability and other measures of autonomic function are warranted to further explore the relationship between autonomic regulation, psychological distress, and cardiovascular risk in MHD patients.

The final model also highlights the multifactorial nature of cardiovascular risk in patients undergoing MHD. Diabetes remained the strongest predictor of MACE in our model, consistent with its well-established role in accelerating atherosclerosis and cardiovascular complications. *Elevated iPTH* was independently associated with increased risk, supporting previous evidence that *disturbances* in mineral metabolism contribute to *vascular calcification* (19) *and myocardial remodeling* (20), and adverse cardiovascular outcomes (21). In contrast, higher UA was inversely associated with MACE (OR = 0.74), which should not be interpreted as evidence of a protective effect. In MHD patients, lower UA may reflect protein-energy wasting, inflammation-malnutrition syndrome, or reduced muscle mass, consistent with the phenomenon of reverse epidemiology. Prior population-based studies have shown that hyperuricemia is associated with endothelial dysfunction and increased cardiovascular risk in the general population (22). However, in dialysis patients, the association between UA and outcomes is likely influenced by nutritional status and residual confounding. Therefore, our findings should be considered hypothesis-generating and warrant further investigation.

The model demonstrated good discrimination (AUC = 0.820), calibration, and clinical utility. A nomogram incorporating RHR, HADS, diabetes, UA, and iPTH enabled individualized risk estimation of MACE risk. Internal validation using bootstrap resampling showed minimal overfitting (optimism-corrected AUC = 0.801), supporting the model’s stability. These findings are consistent with previous risk prediction models in dialysis populations that integrate clinical and biochemical parameters (23), while extending them by incorporating autonomic and psychosocial factors. Given that RHR is routinely measured during dialysis sessions to identify high-risk patients, it may represent a practical component of cardiovascular risk assessment. Nevertheless, external validation in independent cohorts is required before the model can be considered for clinical implementation (24–26).

Several limitations of this study should be acknowledged. First, the absence of systematic assessment of cardiac structure and function represents an important source of unmeasured confounding. Echocardiographic parameters, including left ventricular hypertrophy (LVH) and left ventricular ejection fraction (LVEF), were not for most participants. Given that baseline cardiac status may influence both resting heart rate and cardiovascular outcomes, residual confounding cannot be excluded. Second, this was a single-center study with a relatively modest sample size, which may limit generalizability of the findings to broader maintenance hemodialysis populations. Differences in ethnicity, dialysis practices, and healthcare systems may influence both resting heart rate distribution and cardiovascular risk profiles. Third, resting heart rate was measured only once at baseline. A single measurement may introduce regression dilution bias because resting heart rate varies with volume status, autonomic tone, medication use, and interdialytic changes. Fourth, several clinically relevant confounders were not systematically collected, including smoking status, atrial fibrillation, residual renal function, and detailed assessments of atherosclerotic burden. In addition, the association between uric acid and MACE should be interpreted cautiously because reverse epidemiology is well recognized in maintenance hemodialysis populations. Fifth, the prediction model was developed using logistic regression to estimate fixed-horizon risk rather than to characterize the timing of cardiovascular events; therefore, differences in follow-up duration and event timing were not incorporated into the analysis. Sixth, because BNP measurements and echocardiographic confirmation were not consistently available, some heart failure events were identified based on the treating physician’s documented clinical diagnosis, and a degree of outcome misclassification cannot be excluded. Finally, external validation in independent multicenter cohorts is required before the model can be considered for routine clinical application.

In summary, in this cohort of MHD patients, elevated baseline RHR was independently associated with an increased risk of MACE after adjustment for clinical, metabolic, and psychological factors. A multivariable prediction model incorporating RHR, HADS score, diabetes, UA, and iPTH demonstrated good discrimination and was internally validated. Given its simplicity, low cost, and routine availability, resting heart rate may represent a practical marker for cardiovascular risk stratification in maintenance hemodialysis, although external validation and mechanistic studies are needed to determine whether interventions targeting resting heart rate can improve cardiovascular outcomes in this high-risk population.

## Data Availability

The original contributions presented in the study are included in the article/supplementary material, further inquiries can be directed to the corresponding author/s.
